# V1 as an egocentric cognitive map

**DOI:** 10.1093/nc/niab017

**Published:** 2021-09-14

**Authors:** Paul Linton

**Affiliations:** Centre for Applied Vision Research, City, University of London, Northampton Square, London EC1V 0HB, UK

**Keywords:** V1, retinal map, cognitive map, visual consciousness, predictive coding, multisensory integration

## Abstract

We typically distinguish between V1 as an egocentric perceptual map and the hippocampus as an allocentric cognitive map. In this article, we argue that V1 also functions as a post-perceptual egocentric cognitive map. We argue that three well-documented functions of V1, namely (i) the estimation of distance, (ii) the estimation of size, and (iii) multisensory integration, are better understood as post-perceptual cognitive inferences. This argument has two important implications. First, we argue that V1 must function as the neural correlates of the visual perception/cognition distinction and suggest how this can be accommodated by V1’s laminar structure. Second, we use this insight to propose a low-level account of visual consciousness in contrast to mid-level accounts (recurrent processing theory; integrated information theory) and higher-level accounts (higher-order thought; global workspace theory). Detection thresholds have been traditionally used to rule out such an approach, but we explain why it is a mistake to equate visibility (and therefore the presence/absence of visual experience) with detection thresholds.

## Introduction

Since ‘The Hippocampus as a Cognitive Map’ (O’Keefe and Nadel 1978), we typically distinguish between V1 as an egocentric perceptual map and the hippocampus as an allocentric cognitive map. In this article, we explain why V1 also functions as an egocentric cognitive map.

To the extent that cognitive processing has been discussed in V1, it has focused on (i) the allocation of attention, (ii) top-down influences on perception, and (iii) the transition from egocentric perception to allocentric navigation. By contrast, in this article, we argue that three well-documented functions of V1, namely (i) the estimation of distance, (ii) the estimation of size, and (iii) multisensory integration, are better understood as post-perceptual cognitive inferences. To outline the following sections of this article:

In ‘V1 as a spatial map’, we outline how the field’s understanding of V1 has evolved from a retinal map, to a depth map, to a distance map, to a size map, to a multisensory map.

In ‘V1 as an egocentric cognitive map’, we explain why our recent experimental work suggests that the last three developments, namely the perception of distance, size, and multisensory integration, may be better thought of as purely cognitive in nature and therefore reflect V1 functioning as a cognitive as well as a perceptual map.

In ‘V1 as the neural correlate of the visual perception/cognition boundary’, we consider how V1 could fulfil this dual function by the potential distribution of perceptual and cognitive functions to different layers in V1.

In ‘Low-level theory of visual consciousness’, we explain why this commits us to a low-level account of visual consciousness in contrast to mid-level accounts (recurrent processing theory; integrated information theory) and higher-level accounts (higher-order thought; global workspace theory).

## V1 as a spatial map

In this section, we outline how the field’s understanding of V1 as a spatial map has evolved from a retinal map in the early 20th century, to a depth map in the 1960s, to a distance map in the 1990s, to a size map in the early 2000s, and to a multisensory map in the last couple of decades.

### V1 as a retinal map

V1 provides the initial, and largest, map of the retina in the cortex (Brewer *et al.* 2002; Dougherty *et al.* 2003). Adams and Horton (2003) illustrate just how literally V1 is a retinal map (or a ‘metabolic imprint of the retina’, as they put it) by mapping the shadows cast by retinal blood vessels in V1. The earliest evidence that V1 was retinotopically organized came from Panizza (1855), (Zago *et al.* 2000; Colombo *et al.* 2002), Munk (1881), and Henschen (1890). Building upon pre-existing associations between points on the retina and angular directions in the visual field (Kepler 1604; Descartes 1637; ‘local signs’: Lotze 1846; Hering 1879; 19th-century optometry: Atchison 1979; Johnson *et al.* 2011), Tatsuji Inouye, and then Gordon Holmes, plotted V1’s retinotopic map (Inouye: Inouye 1909; see Glickstein and Whitteridge 1987; Glickstein and Fahle 2000; Leff 2004; Jokl and Hiyama 2007; Leff 2015; Lindegger and Pless 2019; Holmes: Lister and Holmes 1916; Holmes 1918, 1919; see also Riddoch 1917), which was only accurately mapped in the last 30 years (Horton and Hoyt 1991; see Wandell *et al.* 2009; Carandini 2012).

### V1 as a depth map?

Before 1960, it was generally believed that ‘binocular depth perception was based on high-level quasi-cognitive events’ (Bishop and Pettigrew 1986; see also Julesz 1960, 1986) and was therefore processed in higher visual areas. However, disparity selective neurons in V1 were posited by Pettigrew (1965) and found by Barlow *et al.* (1967) and Nikara *et al.* (1968). Although Barlow *et al.* (1967) suggested that this depth information would still have to be ‘sorted out by higher order visual neurones’, they suggested that these disparity selective neurons provided the ‘neural basis of stereopsis’. For a time, it seemed certain that V1 was the site of human stereoscopic depth processing, with Bishop and Pettigrew (1986) distinguishing between ‘Stereopsis before 1960: Mentalism Prevails’ and ‘Retreat from Mentalism begins in the 1960’s’.

However, contemporary reviews of the neural processing of depth perception strike a very different tone (Cumming and DeAngelis 2001; Parker 2007, 2014, 2016; Welchman 2011, 2016; Welchman and Kourtzi 2013; Parker *et al.* 2016; Bridge 2016). According to the literature, responses of binocular neurons in V1 ‘do not reflect perceived depth, but rather compute a local correlation between two images’ (Bridge 2016). So it is now widely accepted that depth perception is ‘extra-striate’ (post-V1) (Cumming and DeAngelis 2001).

### V1 as a distance map

Although V1 is no longer thought of as a depth map, it is still thought to encode viewing distance. As Cumming and DeAngelis (2001) observe ‘Most of the complex questions have not been addressed at the neurophysiological level. One exception is the effect of viewing distance.’ Cumming and DeAngelis (2001) refer to Trotter *et al.* (1992) and Gonzalez and Perez (1998), but the principle dates back to Kepler (1604) and Descartes (1637). The closer an object is, the more the two eyes have to rotate to fixate upon it, and so this degree of eye rotation (or ‘vergence’) provides the visual system with the viewing distance. Trotter *et al.* (1992) found that neurons in V1 were responsive to changes in vergence, and this was confirmed in Trotter *et al.* (1993) and Trotter *et al.* (1996), where they used prisms to change the vergence angle whilst keeping the retinal image and accommodation constant.

Trotter and Celebrini (1999) complete the transition to thinking of V1 in spatiotopic rather than retinotopic terms by showing that half the cells in the monkey V1 are responsive to gaze direction (version eye movements). Importantly, gaze direction (version), when coupled with fixation distance (vergence), marks out a specific location in 3D space, implying that V1 cells ‘are dedicated to certain volumes of visual space’ (Trotter and Celebrini 1999).

### V1 as a size map

Since the early 2000s, it has been suggested that the spatial map in V1 reflects the perceived angular size of objects rather than their retinal size, recasting V1 as a size map rather than a retinal map.

**Figure 1.**
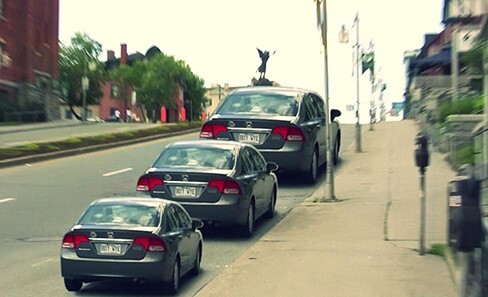
‘Copy-paste, no resize’ by Alex Blouin (https://www.reddit.com/user/weip). © Alex Blouin, reproduced with permission. Original source: https://imgur.com/WBAzkuI For a Reddit discussion of this image see: https://www.reddit.com/r/illusionporn/comments/1mb61j/copypaste_no_resize_xpost_rwoahdude/ And for my discussion, see Linton (2018b).

To explain this distinction, consider Fig. 1. Since the same car is pasted three times, each car in Fig. 1 has the same retinal image size. And yet, the cars appear to be different sizes. The far car appears to take up more of the picture than the near car. This difference in perceived angular size is quantified at around a 20% difference in size (Murray *et al.* 2006; Fang *et al.* 2008). Importantly, Murray *et al.* (2006) found that the activation in V1 reflected the perceived angular size of the stimulus rather than its retinal size. It varied by 20% when participants looked from a near to a far pictorial object, reflecting almost perfectly the change in perceived angular size.

But how is it possible for the V1 to reorganize itself in this way? After all, V1 is supposed to be a retinal map. MacEvoy and Fitzpatrick (2006) hypothesized, and Ni *et al.* (2014) and He *et al.* (2015) found, that V1 receptive fields dynamically re-organize themselves according to the content of the picture (most likely from feedback from lateral occipital cortex (LOC); Zeng *et al.* 2020). This represents ‘a dynamic redrawing of the map of visual space’ (MacEvoy and Fitzpatrick 2006), where specific objects in the picture are seen as magnified or minified (Ni *et al.* 2014).

Another potential size constancy effect is ‘neurological zoom’. This is the suggestion that V1 magnifies the whole visual field with far fixation and minimizes it with near fixation. Marg and Adams (1970) argue that this is responsible for (i) the improvement of visual acuity at far fixation (Aubert and Foerster 1857; Schindel and Arnold 2010), and (ii) changes in the apparent size of after-images with fixation distance (Emmert’s law: Emmert 1881).

Sperandio *et al.* (2012) find that viewing distance modulates V1 activity when observers view an after-image (with Chen *et al.* 2019b finding that the time course suggests recurrent processing of the vergence signal in V1 and/or feedback from higher visual areas). And there is some suggestive evidence of vergence modulating receptive fields in V1 from Cottereau *et al.* (2014) and Marg and Adams (1970).

Sperandio *et al.* (2012) and Chen *et al.* (2019b) suggest that whilst pictorial size constancy is limited to 10–45% (the recent literature suggests around 20%: Murray *et al.* 2006; Fang *et al.* 2008), the scaling of after-images is near perfect because of the additional presence of triangulation cues (vergence, accommodation, binocular disparity, and motion parallax).

However, Chen *et al.* (2019a) were surprised to find that although their subjects experienced close to perfect size constancy in real-world viewing conditions, recorded neural activity could only account for half of this effect. The problem is that Sperandio *et al.* (2012), Chen *et al.* (2019b) and Chen *et al.* (2019a) all overlook the distinction between perceived physical size and perceived angular size. It is true that participants may judge the perceived physical size of objects almost perfectly in real-world viewing conditions. But that does not mean that the perceived angular size of an object never reduces with distance. There are two effects at play. First, angular size: Does the perceived angular size simply reflect the retinal image size or does it also incorporates perceptual effects (‘size constancy’)? Second, physical size: Our ability to judge the constant physical size of an object despite a reduction in its perceived angular size with distance. This is clearly a cognitive effect since it makes up whatever shortcomings there are in our perception.

### V1 as a multisensory map

In the last couple of decades, there has been increasing evidence of V1 as the site of multisensory integration, with Murray *et al.* (2016) observing that ‘convergence and integration of information from different senses within low-level cortices is a rule rather than an exception (e.g. Ghazanfar and Schroeder 2006; van Atteveldt *et al.* 2014; De Meo *et al.* 2015; ten Oever *et al.* 2016)’. We find evidence of multisensory integration in V1 in three specific contexts:

#### Vision and sound

The quote from Murray *et al.* (2016) comes from audio–visual integration, where there is evidence of bottom-up signals travelling from the auditory cortex to V1 (see Falchier *et al.* 2002; Rockland and Ojima 2003 on monkeys; Beer *et al.* 2011 on humans).

In human fMRI, Calvert *et al.* (2001) find increased activation in V1 when a visual stimulus is accompanied by a synchronous sound, whilst Martuzzi *et al.* (2007) find that sound alone can activate V1. Watkins *et al.* (2006, 2007) studied the sound-induced flash illusion and find increased activation in V1 when one visual flash and two auditory beeps are experienced as two visual flashes. Studies also demonstrate that sound can modulate the orientation preference of neurons in layers 2/3 (Ibrahim *et al.* 2016; Chanauria *et al.* 2019; McClure and Polack 2019) and 5/6 (Chanauria *et al.* 2019) of V1, but not layer 4 (Ibrahim *et al.* 2016; Chanauria *et al.* 2019), emphasizing that we should not think of V1 as a single map, but instead consider the functional role of each layer (a point developed below).

In addition to these bottom-up effects, there is also evidence of top-down contextual effects (Petro *et al.* 2017). Vetter and colleagues show that the high-level semantic categories (‘bird singing’, ‘people talking’, and ‘traffic’) can be decoded from V1 in blindfolded (Vetter *et al.* 2014) and congenitally blind (Vetter *et al.* 2020) participants. By contrast, although Masuda *et al.* (2021) also find task-dependent signals in V1 in the blind visual field of participants with macular degeneration, these signals are not detectable in control participants, leading them to question the extent to which these top-down signals have an impact on normal visual processing in V1. Masuda *et al.* (2021) conclude that ‘top-down, modality independent signals are ordinarily suppressed by feedforward signals and become profound in the absence of retinal inputs.’

#### Vision and touch

Masuda *et al.* (2021) come to the same conclusion for the integration of vision and touch. Whilst they find touch signals are present in the blind visual field of participants with macular degeneration during active tasks, these signals are neither present when passively applied, nor are they found in normally sighted controls, suggesting that they merely reflect ‘task-related feedback signals rather than reorganized feedforward visual inputs.’ Similarly, Couto *et al.* (2019) found that neurons in the mouse V1 failed to respond to tactile stimuli when the mouse’s whiskers were passively stroked, suggesting that the impact of other modalities on V1 is restricted to modality-independent top-down effects.

Sadato *et al.* (1996) did find activation of V1 during braille reading by blind participants. However, this does not appear to reflect typical multisensory integration, so much as the recruitment of V1 for touch after a period of visual deprivation (Merabet *et al.* 2008; Bedny 2017). On the other hand, Merabet *et al.* (2004) did find a small effect of transcranial magnetic stimulation to V1 on sighted participants’ ability to discriminate the spacing of a row of dots felt by touch, potentially reflecting a compromised ability to assign spatial attention (Macaluso *et al.* 2000).

#### Vision and locomotion

The perception of self-motion relies on integrating retinal stimulation (optic flow) with signals about bodily motion, so this is a prime candidate for multisensory integration in V1.

We can distinguish between passive self-motion and active self-motion. In passive self-motion, Roberts *et al.* (2017) find increased activation in V1 in human participants when visual and vestibular cues are congruent, but no effect when they are incongruent. Roberts *et al.* (2018) also find that vestibular neuritis patients (who typically suffer from acute vertigo from impaired vestibular function) have reduced V1 activation in congruent conditions.

However, much of the recent attention has been on active self-motion, specifically in mice (for reviews see Busse *et al.* 2017; Pakan *et al.* 2018; Wang *et al.* 2020; Saleem 2020). Niell and Stryker (2010) found increased firing in V1 with motion, and Keller *et al.* (2012) and Saleem *et al.* (2013) found that running in darkness activates V1. Ayaz *et al.* (2013) also found that motion reduces surround suppression, enabling V1 neurons to integrate over larger regions of visual space.

In terms of multisensory integration, Saleem *et al.* (2013) found that most of the V1 neurons that responded encoded a weighted sum between optic flow and running speeds. Furthermore, Keller *et al.* (2012) found that layer 2/3 of V1 is strongly modulated by a mismatch between actual and expected optic flow, leading them to suggest that V1 is engaged in predictive coding (Rao and Ballard 1999; Koch and Poggio 1999; Friston 2018), with Zmarz and Keller (2016) finding that this occurs locally at the level of the receptive fields of individual neurons (for further discussion of predictive coding of optic flow in V1 see Leinweber *et al.* 2017; Fridman and Petreanu 2017; Keller and Mrsic-Flogel 2018; Jordan and Keller 2020; Padamsey and Rochefort 2020).

Increasingly, V1 has been found to include the location of objects in the visual scene and their behavioural relevance (see Pakan *et al.* 2018; Saleem 2020; Flossmann and Rochefort 2021). Ji and Wilson (2007) found that during sleep V1 replayed the same experience (presumably as dreams) as the hippocampus. Poort *et al.* (2015) found that perceptual learning led to an increase in the response of layer 2/3 to a visual stimulus, and Fiser *et al.* (2016) found different responses to the same visual stimulus depending on where it was encountered in the environment, with both studies also finding neurons that anticipated the stimulus. There is increasing evidence that these responses are due to top-down feedback from the anterior cingulate cortex and likely also the retrosplenial cortex (Fiser *et al.* 2016). Pakan *et al.* (2018b) find that the layer 2/3 neurons that encode spatial position also respond to self-motion defined estimates of distance when visual cues are absent and give their results a predictive coding explanation (Pakan *et al.* 2018a).

Other studies that report subjective position in V1 include Saleem *et al.* (2018) and Diamanti *et al.* (2020) (in layer 2/3) and Fournier *et al.* (2020) (in layers 4/6), but they have a slightly different focus. Pioneering parallel recording in V1 and the hippocampus, they ask how we get from egocentric (vision, V1) to allocentric (navigation, hippocampus) models of the environment? Because Fournier *et al.* (2020) find that the effect of subjective estimates of position is reduced around visual landmarks, they suggest that V1 plays an early role in navigation by recalibrating estimates of the distance travelled when a salient visual landmark is passed.

## V1 as an egocentric cognitive map

The last section on multisensory integration demonstrates increasing evidence of non-visual top-down processing in V1. What is this processing for? We outline the three explanations in the literature before advancing our own proposal that, in addition to a retinal map, V1 acts as a post-perceptual egocentric cognitive map.

### Anchoring the hippocampus’ cognitive map

We classically draw a distinction between V1 as an egocentric visual map and the hippocampus as an allocentric cognitive map (Tolman 1948; O’Keefe and Nadel 1978, 1979). As we just saw, one explanation for non-visual processing in V1 is that V1 plays an early role in calibrating the allocentric cognitive map (navigation, hippocampus) to our current egocentric visual experience (perception, V1) (see also Nau *et al.* 2018). This is one facet of a very active research question that seeks to explain how we ‘anchor the cognitive map to the visual world’ (Julian 2017), with others also pointing to egocentric, as well as allocentric, frames of reference in the hippocampus as another potential mechanism (see Wang *et al.* 2020 for a review). The key thing to recognize is that this research agenda maintains the classic distinction between egocentric (vision, V1) and allocentric (cognitive map, hippocampus) models of the environment. The underlying assumption of this line of research is that visual perception is egocentric and spatial cognition is allocentric, and the question is how we translate between them.

### Cognitive penetration

There are two paradigms in the multisensory literature (Couto *et al.* 2019). The first treats multisensory integration in V1 as bottom-up, and horizontal, rather than hierarchical (Watkins *et al.* 2006). However, given the prevalence of top-down multisensory signals reviewed in the last section, this leads to the suggestion that bottom-up processes in V1 are subject to top-down ‘cognitive penetration’ (Vetter and Newen 2014; Newen and Vetter 2017). What this means is that these top-down signals are influencing our visual experience. Take the findings by Poort *et al.* (2015) and Fiser *et al.* (2016) that expectations lead to an increased response in V1. Poort *et al.* (2015) suggest that this provides the observer with ‘enhanced and more distinct representations of task-relevant stimuli’, and Fiser *et al.* (2016) suggest that it works to ‘enhance the discriminability of similar stimuli in different contexts.’

### Predictive processing

The second, more radical, but increasingly orthodox paradigm in the multisensory literature treats perception as one large predictive process (Rao and Ballard 1999; Keller and Mrsic-Flogel 2018; Petro *et al.* 2017; Newen and Vetter 2017 also frame their work in these terms). On this account, perception is hierarchical, and the perception/cognition distinction is effectively eradicated. This is consistent with predictive (Bayesian) models of visual perception (Knill and Richards 1996; Trommershauser *et al.* 2011; Frith 2013) according to which (at least on one popular interpretation) we see what we predict.

### Post-Perceptual cognitive processing

Both ‘cognitive penetration’ and ‘predictive processing’ are committed to the idea that top-down signals to V1 influence our visual experience. With the exception of the suggestion that V1 provides the early stages of anchoring the hippocampus’ cognitive map, it is a truism of the literature that V1 processing equates with visual processing. By contrast, we argue that V1 is the site of egocentric spatial cognition, as well as egocentric spatial perception.

The account of visual perception I develop in Linton (2017) (reviewed by Erkelens 2018, and elaborated in Linton 2018f, 2021a; the *Brains Blog*: Linton, 2018a; 2018b, 2018c, 2018d, 2018e and two experimental papers: Linton 2020, 2021b) argues that many of the supposedly basic aspects of visual perception are better understood as post-perceptual cognitive influences. This includes (i) visual scale (the size and distance of objects; see Linton 2017, pp.134–136, 2018f), (ii) visual shape from multiple depth cues (depth cue integration; see Linton 2017, chapter 2), (iii) pictorial cues and pictorial perception (Linton 2017, chapter 3), and (iv) multisensory integration (Linton 2017, pp.65–66). However, as Erkelens (2018) notes, a ‘shortcoming of the book is that … it does not discuss the new theory in relation to the neurophysiology of the visual system.’ The purpose of this article is to rectify this shortcoming.

Given our survey of V1 in Section 1, the implications of my theory for the neurophysiology of the visual system become apparent. On one hand, as we saw in Section 1, both visual scale (the size and distance of objects) and multisensory integration are now regarded as paradigmatic V1 processes. On the other hand, under my account, both visual scale (the size and distance of objects) and multisensory integration are automatic post-perceptual cognitive inferences. This leads us to the conclusion that, in addition to V1 functioning as an egocentric perceptual map, V1 must also be functioning as a post-perceptual egocentric cognitive map.

In the rest of this section, I will focus on substantiating this claim. But before I do, I need to explain what I mean by ‘automatic post-perceptual cognitive inferences’.

### Perception/cognition distinction

We mark the ‘perception’/‘cognition’ boundary as the point at which mental processes become ‘about’ our perceptual experience rather than ‘contributing to’ our perceptual experience. We define ‘perceptual experience’ as experience in any sensory modality; for instance, visual experience (what we see), auditory experience (what we hear) and tactile experience (what we feel through touch). And the focus of Linton (2017) is to try to understand which visual cues contribute to our visual experience, as opposed to merely influencing our cognition or understanding of our experience.

To make this distinction clearer, we consider the example of reading. We argue that two people have the same visual experience of words on a page if one can read and the other cannot, even though they have very different cognitive experiences of them. In this way, being able to read changes our experience of a visual stimulus, without changing our visual experience of the stimulus (understanding or attributing meaning often has an experiential quality, see Strawson 1994; Bayne and Montague 2011 on ‘cognitive phenomenology’). We believe that reading captures a common intuition about the ‘perception’/‘cognition’ distinction. Defining reading as ‘perceptual’, as opposed to ‘cognitive’, would appear to define ‘perception’ too broadly. Indeed, one wonders what, if any, automatic cognitive process that relies on visual input (driving, navigation, etc.) would be excluded by such a broad definition of ‘perception’.

We apply the thought about reading to the interpretation of signs more broadly, which covers associations as diverse as understanding that there is fire by seeing smoke, understanding when to stop and when to go at a traffic light, understanding hand gestures, and understanding symbols like the McDonald’s logo (Atkin 2013). This attribution of meaning through association is, we argue, paradigmatic of post-perceptual cognitive processes. So, under our definition, work in computer vision based on object recognition in images (Tarr and Bulthoff 1998), as well as ventral stream processing that instantiates object recognition in the human cortex (Goodale and Milner 1992), should be thought of as cognitive rather than perceptual.

In Linton (2017), we go still further and argue that many apparently basic aspects of visual perception (size and distance, depth cue integration, multisensory integration, and pictorial cues and pictorial perception) are better thought of as automatic post-perceptual cognitive inferences about our visual experience, rather than contributing to our visual experience. Ultimately, this is an empirical question—we cannot rely on our introspection to tell us whether a process contributes to our visual experience or not—and so the focus of this section is on presenting our empirical data that suggest that a number of well-studied V1 processes are purely cognitive in nature.

This is a broad definition of ‘cognition’. Cognition can be unconscious (we are unaware of it at work), automatic (we do not have to do anything), and involuntary (we often cannot overrule it). Most cognitive scientists accept a broad definition of ‘cognition’ (see Heyes in Bayne *et al.* 2019; ‘System 1’ vs. ‘System 2’ in Kahneman 2011). And this was how ‘cognition’ was conceived during the ‘cognitive revolution’ of the 1960s, a term Neisser (1967) coined to ‘do justice … to the continuously creative process by which the world of experience is constructed.’

“Visual cognition … deals with the processes by which a perceived, remembered, and thought-about world is brought into being from as unpromising a beginning as the retinal patterns.” Neisser (1967)

However, unlike most cognitive scientists, I do not believe these ‘mid-level’ processes contribute to our visual experience. In Linton (2017), I therefore draw a sharp distinction between my own approach and contemporary approaches to ‘visual cognition’ (Cavanagh 2011). Again, this is not necessarily counter-intuitive. The more ‘mid-level’ processes we add to vision, the more we open the door to the suggestion that (in Cavanagh 2011‘s words) ‘our visual systems, on their own, rank with the most advanced species in cognitive evolution.’

“...the unconscious inferences of the visual system may include models of goals of others as well as some version of the rules of physics. If a ‘Theory of Mind’ could be shown to be independently resident in the visual system, it would be a sign that our visual systems, on their own, rank with the most advanced species in cognitive evolution.”

And, by equating ‘vision’ with any automatic process that relies on visual input, we go far beyond visual experience. For instance, in Glennerster (2016) ‘the visual system’ is now responsible for performing our everyday tasks, such as getting a mug from another room:

“The visual system must somehow know that the mug is behind one door rather than another, even if it does not store the three-dimensional location of the mug.”

This is the problem we lead ourselves into by identifying every visually informed unconscious inference or automatic process as ‘visual perception’.

In contrast to my negative (‘not visual experience’) definition of cognition (Linton 2017; see also Brainard in Bayne *et al.* 2019), most philosophers adopt a positive and high-level definition of ‘cognition’ (e.g. Bayne in Bayne *et al.* 2019; see Heyes in, 2019). This is typically what people mean when they ask whether ‘cognition’ penetrates ‘perception’ (Pylyshyn 1999; Firestone and Scholl 2016). But this definition of ‘cognition’ is liable to distort discussions. For instance, Knotts *et al.* (2020) describe automatic inferences about perception as ‘intra-perceptual’ rather than ‘post-perceptual’ or ‘cognitive’ because automatic inferences are immune from ‘cognitive penetration’. But this new category (‘intra-perceptual’) risks conflating our biased post-perceptual judgements about our perceptual experience [when ‘subjective evaluation of (our visual experience) is systematically biased, or inflated’] with perceptual experience itself, both of which may be immune to ‘cognitive penetration’.

The essential question of the ‘perception’/‘cognition’ distinction is whether a process contributes to our visual experience or not? (Linton 2017; Brainard in Bayne *et al.* 2019; a point Abid 2019 makes well in response to Odegaard *et al.* 2018). Knotts *et al.* (2020) appear to suggest that the answer to this question in the context of ‘intra-perception’ is ‘yes’, in which case it is hard to see why we need a new category, in addition to ‘perception’.

Knotts *et al.* (2020) give three examples of ‘intra-perceptual’ biases: (i) the Müller–Lyer illusion, (ii) multisensory integration, and (iii) amodal completion. But all three examples seem to fit quite neatly within the existing ‘perception’/‘cognition’ distinction. Most vision scientists would agree with Knotts *et al.* (2020) that both the Müller–Lyer illusion and multisensory integration affect our visual experience, and this is why most scientists would refer to them as ‘perceptual’. By contrast, I argue that the Müller–Lyer illusion and multisensory integration do not affect our visual experience, and this is why I refer to them as merely ‘cognitive’ (Linton 2017, pp.60–66, 2018b). Amodal completion is an interesting case. I agree with Knotts *et al.* (2020) that many would regard amodal completion as ‘perceptual’. However, I disagree with Knotts *et al.* (2020) that the borders of the occluded object are ‘still visually represented’ in amodal completion. As Knotts *et al.* (2020) themselves admit, amodal completion is merely just ‘a feeling’. In which case, it is merely an automatic cognitive inference about our percept and fits naturally within ‘cognition’. Note also that Bergmann *et al.* (2019) were unable to decode amodal completion in V1, suggesting that this effect is very much a purely cognitive process.

Having outlined how I draw the distinction between ‘perception’ and ‘cognition’, let me now substantiate why I believe that visual scale (size and distance) and multisensory integration fall on the ‘cognition’ side of the distinction, with an emphasis on V1 processing.

### V1 as a cognitive distance map

You will recall that the viewing distance is instantiated in V1 using vergence eye movements (Trotter *et al.* 1992) so that ‘the visuo-oculomotor transformation process appears to start as early as the primary visual cortex’ (Trotter *et al.* 1993).

**Figure 2.**
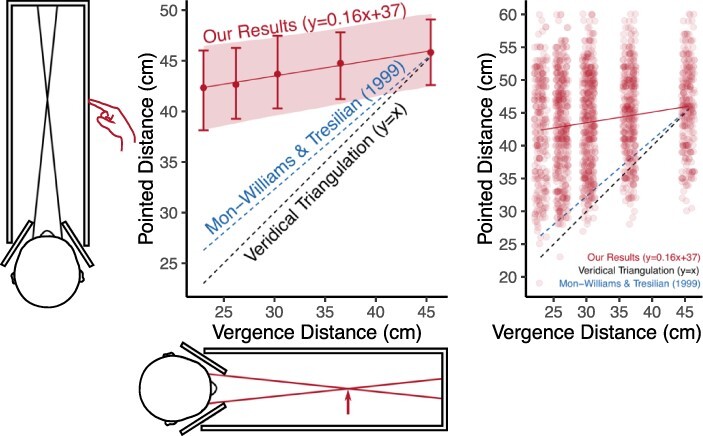
Results from experiment 2 in Linton (2020). Mon-Williams and Tresilian (1999) represents how accurate vergence is traditionally assumed to be. My setup was very similar to theirs, but in Mon-Williams and Tresilian (1999) the changes in vergence were sudden rather than gradual that gave participants subjective knowledge about their changing eye movements. In my experiment, vergence was manipulated gradually by increasing or decreasing the separation between two targets on a screen. Metal plates on either side of the observer’s head ensured that the right eye only saw the left target and the left eye only saw the right target, enabling us to manipulate the vergence distance (where the two lines of sight intersect, indicated by the arrow). Participants then had to the point to the distance of the target using a non-visible hand. © Paul Linton (CC-BY-4.0).

But is this a ‘perceptual’ or ‘cognitive’ process? Applying the ‘perception’/‘cognition’ distinction we outlined above, the question is whether eye movements really affect our visual experience? One way to test this is to vary vergence gradually, and see if vergence still has an effect on our visual experience and, therefore, on our distance judgements, even when participants are subjectively unaware that their eyes have moved. In Linton (2020), I tested this by gradually manipulating vergence and found that participants were unable to use their visual experience to judge distance. Instead, they were effectively guessing (Fig.2).

These results are complemented by our results in Linton (2021b) on size perception (discussed below). Together they pose a real challenge to the suggestion that eye movements affect our visual experience. This work has two important implications.

First, we are not challenging Trotter *et al.* (1992)’s finding that vergence is encoded in V1. However, since vergence is encoded in V1 and since vergence does not appear to affect our visual experience, we conclude that V1 must be cognitively (and not perceptually) encoding egocentric distance from vergence. V1 might be encoding another distance cue that does affect our visual experience. However, the point developed in Linton (2018f) and Linton (2020) is that all of our other absolute distance cues are either similarly ineffective (absolute motion parallax, vertical disparities) or merely cognitive (familiar size) in nature. In any case, this is not essential to the point since Trotter *et al.* (1992) specifically test absolute distance signals in V1 in reduced cue conditions in order to isolate the vergence signal from other absolute distance cues.

Second, if vergence (eyes moving in the opposite direction) does not affect our visual experience, we have to question whether version (eyes moving in the same direction) does? Version eye movements are thought to be essential for visual direction (in order to convert retinotopic coordinates into spatiotopic coordinates). So, unless eye movements affect our visual experience, we are forced to conclude that visual direction is also merely cognitive. This will be the focus of future experiments. It also opens up the question of whether retinal stability is merely cognitive. This is because the visual system is making roughly three saccades per second (Yarbus 1967). So unless eye movements affect our visual experience (Keller and Mrsic-Flogel 2018), the stabilization of the visual scene over time would have to be merely cognitive as well.

### V1 as a cognitive size map

What about the size constancy effects reported in V1. Are they ‘perceptual’ or merely ‘cognitive’? You’ll recall that Sperandio *et al.* (2012) draw a distinction between size constancy (i) in the real world (which, according to Sperandio *et al.* 2012 is close to perfect) and (ii) in pictures (which is around 20%; Murray *et al.* 2006).

#### Real-world size constancy

Sperandio *et al.* (2012) suggest that in full cue conditions their fMRI results show that V1 represents the perceived rather than retinal size of the object. Size constancy is close to perfect, they argue, because in addition to pictorial cues, in real-world viewing, participants have access to additional depth cues including vergence, binocular disparity, and motion parallax. However, the participants in Sperandio *et al.* (2012) did not have motion parallax (they were in an fMRI scanner), and binocular disparity is thought to primarily rely on vergence to provide absolute distance information (Wallach and Zuckerman 1963). So it is no surprise that a central question is how reliant we are on vergence for size constancy.

A key site for this discussion is the Taylor illusion (Taylor 1941). Here, participants view an after-image of their hand (which fixes the retinal size of their hand) in complete darkness, and then move their hand forward or backward in space. Taylor (1941) found that participants report that the hand appears to shrink when brought close to the face and expand when moved away. The accepted explanation of the Taylor illusion is that it is entirely due (Taylor 1941; Morrison and Whiteside 1984; Mon-Williams *et al.* 1997) or almost entirely due (Sperandio *et al.* 2013) to changes in vergence as the eyes track the hand in darkness (see also Gregory *et al.* 1959; Carey and Allan 1996; Bross 2000; Ramsay *et al.* 2007; Faivre *et al.* 2017a and on vergence scaling; Urist 1959; Suzuki 1986; Lou 2007; Zenkin and Petrov 2015).

However, the problem with these experiments is that they rely on manipulating the vergence angle in a way that informs the participant about the change in fixation distance, by asking participants to track their own hand as it moves. These experiments are therefore open to an alternative cognitive explanation, namely that changes in vergence do not affect the perceived angular size of the hand but merely affect our cognitive understanding of its size given its fixed angular size and our subjective knowledge about our changing fixation distance.

In order to differentiate between these two interpretations, we replicated the vergence changes in Sperandio *et al.* (2013) (distance change from 50 to 25 cm over 5 seconds) but this time controlling for any cognitive knowledge about the change in distance by having participants track a visual target rather than an light-emitting diode (LED) or their hand. Tracking their own hand clearly gives participants knowledge about their changing gaze position. So too does an LED moving in depth, because the eyes are very bad at tracking a single point moving in depth, leading to retinal slip (motion of the LED on the retina). By contrast, we used a 3° target to ensure that any retinal slip from the target moving in depth was undetectable. We found no evidence that vergence affects perceived size once subjective knowledge about the changing gaze position has been controlled for (Linton 2021b). We therefore conclude that the vergence size constancy effects reported in the literature are merely cognitive rather than perceptual.

#### Size constancy in pictures

**Figure 3.**
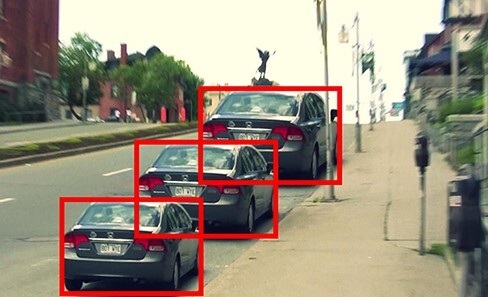
Amended version of Fig. 1 from Linton (2018b) with equal sized rectangles.

What about the pictorial size constancy in Murray *et al.* (2006)? There is no denying that the car at farther distance seems to take up more of the picture than the near car in Fig. 1. But a key point of Linton (2017) is that we cannot rely on introspection to tell us if an effect is perceptual or merely cognitive. Instead, a useful strategy is what we call ‘scaffolding’: introducing elements into the visual scene that help us to judge what we are seeing. In Linton (2018b), we apply this logic to pictorial size constancy, as well as the Müller–Lyer illusion. The claim in the literature, as Schwarzkopf (2015) rightly points out, is that pictorial size constancy works by ‘distorting the entire map of visual space through shifting the receptive fields as suggested by electrophysiological recordings.’ But if this is right, and pictorial size constancy really does distort visual space, by enlarging some regions and shrinking others, then it should also equally distort any additional pictorial elements introduced into the picture. However, consider Fig. 3, an amended version of Fig. 1 from Linton (2018b).

According to the logic of the literature, the squares that bound the exact same area in visual space as the cars should be just as distorted in size as the cars. But this is not what we experience in Fig. 3. Instead, the size difference, if any, between the squares is vastly reduced. So what we took to be a perceived change in size between the cars is actually entirely (our position), or almost entirely (if you insist there is a small difference in size between the squares), a cognitive effect.

### V1 as a cognitive multisensory map

This way of thinking about the ‘perception’/‘cognition’ distinction also leads us to reinterpret multisensory integration as cognitive rather than perceptual. The central question is whether multisensory integration actually affects our visual experience, rather than merely our interpretation of it. And we appeal to four specific examples to argue that it does not.

#### Mandatory fusion

In Linton (2017), we argue that different kinds of visual depth cues (pictorial cues and binocular disparity) are not perceptually integrated, but merely cognitively integrated. This is because the experiments that test visual cue integration (Hillis *et al.* 2002) test the stimuli in highly artificial conditions conducive to visual cues being judged to be perceptually fused when they are not. And, if merely cognitive integration explains the integration of visual cues, then we would also expect it to explain less immediate effects like multisensory integration.

But you do not need to accept my analysis of visual cue integration to come to this conclusion. If two cues are perceptually integrated, you would expect a change in one of them in one direction to cancel out a change in the other in the other direction. This is known as ‘mandatory fusion’. But Hillis *et al.* (2002) find that unlike visual cues, multisensory integration (between vision and touch) is not subject to ‘mandatory fusion’. Similarly, failures in ‘mandatory fusion’ have been found in vision and sound (Caziot and Mamassian 2017) and vision and vestibular cues (Ash *et al.* 2011). This raises the following dilemma (Linton 2018a). Where, perceptually, are these multisensory percepts experienced? Not in vision, which reports the visual estimate. And not in touch, which reports the touch estimate. So there does not appear to be a sensory experience where we actually experience the multisensory integration. It does not change our visual experience, and it does not change our tactile experience. So where is it? (Contrast this with the suggestions in the next paragraph that visual experience ‘relies on multimodal signals’ and that ‘visual consciousness is shaped by the body’). Instead, we conclude (Linton 2017, p.66) that ‘the absence of mandatory fusion in … cross-modal perception appears to suggest that when information is integrated in these contexts it is by virtue of an *integrated judgement* rather than an *integrated percept*.’

#### Taylor illusion

Our approach to vergence eye movements provides a model for multisensory integration more generally. Sperandio *et al.* (2013) find that hand movements, and not just the change in vergence, directly affects the perceived size of the after-image of the hand in the Taylor illusion. They demonstrate this by showing that the strength of the illusion is marginally reduced when vergence and the hand move in opposite directions. Consequently, the Taylor illusion is thought to provide evidence for the suggestion that vision ‘relies on multimodal signals’ (Sperandio *et al.* 2013; Chen *et al.* 2018) and that ‘visual consciousness is shaped by the body’ (Faivre *et al.* 2015, 2017a,b). But if, as we argue in Linton (2021b), the integration of the retinal image with the proprioceptive sensation of our own vergence eye movements is purely cognitive (and this is the major driver of the Taylor illusion), then it becomes very likely that the integration of the retinal image with the proprioceptive sensation of our own hand movements is also purely cognitive when this is a minor driver of the Taylor illusion. This cognitive interpretation of the influence of hand movements on visual perception also suggests a cognitive interpretation of variants of the Taylor illusion that show multisensory integration with the rubber-hand illusion (Faivre *et al.* 2017a) and tool use (Grove *et al.* 2019).

#### Rubber-hand illusion

The rubber-hand illusion (Botvinick and Cohen 1998) poses another question. How is it that visual and tactile sensations can be spatially decoupled in this way? The traditional explanation is that the signal from each sense is corrupted by noise (Trommershauser *et al.* 2011), so each signal is liable to give different spatial estimates. But the degree of decoupling involved in the rubber-hand illusion far exceeds what one would expect from two identical, but noisy, estimates of the same spatial location. Instead, in Linton (2018f), we argue that vision and touch can come apart in this way because they do not share a common metric. Put simply, they are not giving conflicting spatial information because they don’t operate in the same space. Without eye movements influencing visual perception, vision is retinotopic not spatiotopic (we put to one side the question of whether touch is head-centric or body-centric; if the latter, a further conversion between head-centric and body-centric coordinates would be required for vision). So without eye movements influencing visual perception, vision does not provide us with absolute distance or direction. Touch, we presume, does. And so a further, cognitive, step is required to convert vision into the same metric as touch so that they can be integrated. But this further, cognitive, step is subject to significant error. (Contrast this account with a very different cognitive interpretation of the rubber-hand illusion by Lush *et al.* 2020; Roseboom and Lush 2020, according to which there is no integration, perceptual or cognitive).

#### Self-motion

Finally, at the conceptual level, why should we expect multisensory integration to be perceptual? In Linton (2018f), we discuss this thought in the context of the integration of visual (optic flow) and vestibular cues to passive self-motion. Adding vestibular cues to optic flow increases the impression of self-motion over and above optic flow alone (Ash *et al.* 2011). But why would we expect this to affect our visual experience? It is not as if the optic flow has to be seen as flowing faster in order to incorporate this vestibular cue into our determination of self-motion. Instead, the visual (optic flow) and the non-visual (vestibular) cues merely feed into a common post-perceptual cognitive determination of speed.

## V1 as the neural correlate of the visual perception/cognition boundary

We therefore argue that V1 is the site of the visual perception/cognition distinction, acting both as (i) the site of visual experience and (ii) where automatic post-perceptual inferences about our own visual experience occur.

Our suggestion of V1 as a cognitive map is a very different from Zhaoping (2014, 2019)’s description of V1 as a ‘saliency map’. Zhaoping (2019)’s ‘saliency map’ is concerned with attributing cognitive meaning (saliency) to the scene prior to perception in order to drive eye movements to specific meaningful locations in the scene. This is because, under Zhaoping (2019) account, ‘V1 activities are unrelated to visual awareness’, and so fixational eye movements are required to pass the stimulus via attention to higher visual areas for visual awareness. Under Zhaoping (2019) account, (i) V1 is pre-perceptual, and (ii) Zhaoping (2019) ‘saliency map’ is a pre-perceptual ‘cognitive map’ of sorts. By contrast, we are arguing that (i) V1 is the site of visual experience (V1 as visual map), and also (ii) V1 is also the site of the first layer of post-perceptual cognition (V1 as cognitive map).

But how can V1 be both a visual map (providing our visual experience) and also a post-perceptual cognitive map (interpreting our visual experience), at the same time? We suggest that the answer lies in the laminar structure of V1. It is typical to distinguish between superficial (2/3), mid (4), and deep (5/6) layers of V1. Visual inputs from LGN feed primarily into layer 4C, so layer 4 would appear to be most likely area for ‘pure’ binocular visual experience divorced from cognitive inferences (cf. Fournier *et al.* 2020 on navigation; Self *et al.* 2013 on figure/ground; and, given my comments on motion below, Livingstone and Hubel 1988).

Differentiating between layers of V1 may also help us to explain why our visual experience does not appear to be high-pass spatially filtered, whilst this is regarded as a key aspect of V1 visual processing (Carandini 2012). For instance, if high-pass spatially filtering were primarily an aspect of layer 2/3 (feeding into higher visual areas), but the inputs at layer 4 remained unfiltered, and were experienced as visual perception, then this would provide a convincing explanation of why spatial filtering is a key aspect of V1 visual processing, and yet is not directly experienced.

Since higher visual areas predominantly feed into layer 2/3 and more distant cortical areas predominantly feed into layer into 5/6, one suggestion is that top-down influences to layer 2/3 are reflected in illusions that we experience as visual perception, whilst top-down influences to layer 5/6 are reflect as imagery (Bergmann *et al.* 2019; Koenig-Robert and Pearson 2021). One potential problem with this account is that Kok *et al.* (2016) find that the Kaniza triangle illusion only activates layer 5/6, not 2/3. However, it could be that the Kaniza triangle really is better thought of as a form of involuntary visual imagination (consistent with evidence in Kok *et al.* 2016 of attendant suppression of perception in layers 2/3 and 4), although we continue to prefer a purely cognitive interpretation of the Kaniza triangle (Linton 2017, p.11).

However, our key concern with Bergmann *et al.* (2019)’s and Koenig-Robert and Pearson (2021)’s suggestion that we experience layers 2/3 as visual perception, and layers 5/6 as imagery, is that visual inputs also affect layer 5/6, as if imagery and perception are being integrated. Instead, we regard one of two solutions as the most likely interpretation.

The simplest solution would be to regard layer 4 as the site of conscious visual experience (which we describe above as ‘pure’ binocular visual experience) and layers 2/3 and 5/6 as two different layers of cognitive processing. This is supported by Jordan and Keller (2020)’s finding of two different interactions between visual and motor signals in V1: comparison in layers 2/3 (for visual stability) and integration in layers 5/6 (for the integrated ‘percept’) (both of which I would give a cognitive interpretation) (see also Keller and Mrsic-Flogel 2018; Padamsey and Rochefort 2020). Further support comes from Self *et al.* (2013) on the time course of figure-ground segmentation in layers 2/3 and 5/6.

Another approach, which we also have some sympathy for, would be to regard layers 5/6 as the site of visual experience, over which bottom-up perception and top-down imagination compete, with dreams resulting when imagination wins that competition globally, and hallucinations resulting when imagination wins that competition in a local region of space.

Whichever interpretation is correct, the key point is that somewhere feedforward visual processing is dominant, and this is reflected as our visual experience.

## Low-level theory of visual consciousness

If V1 is the first site of post-perceptual cognitive processing, then it must logically be the site of visual experience as well (unless you want to suggest that visual experience is situated prior to V1 in LGN, which is a conceptual possibility, but which V1 acting as the site of binocular integration appears to rule out).

Thinking of V1 as the site of visual experience provides some potentially helpful insights about the nature of visual ‘illusions’ which, because we give them a cognitive interpretation, are ‘better thought of as delusions (false judgements) rather than illusions (false percepts)’ (Linton 2017, pp.8, 69, 74). First, as we have already noted, Bergmann *et al.* (2019) were unable to decode amodal completion in V1, suggesting that this effect is very much a purely cognitive process. Second, Bergmann *et al.* (2019) were only able to decode the edges of the neon spreading illusion in V1. But the neon spreading illusion is of a single illusory surface. This suggests that the illusory surface in the neon spreading illusion is also cognitive rather perceptual.

Distinguishing between the layers of V1 (and arguing that feedforward processing in layer 4 reflects visual perception, whilst feedback processing from higher visual areas into layers 2/3 merely reflects cognition) leads us to embrace a low-level theory of visual consciousness, in contrast to mid-level theories (recurrent processing theory; integrated information theory) and higher-level theories (higher-order thought; global workspace theory). This is important, because most of the recent adversarial collaborations in consciousness science today have focused on adjudicating between mid- and higher-level approaches, whilst missing this third distinct alternative (Templeton grants 0567: He, Peters, Denison, Brascamp, Block, & Chalmers, ‘An adversarial collaboration to test predictions of first-order and higher-order theories of consciousness’, 2020−2023; 0484: Block, ‘Analyzing and merging theories of consciousness’, 2020−2023; and 21569: Block & Lau, ‘Arbitrating philosophical theories of consciousness by cognitive neuroscience experiments’, 2011−2015). Let me explain the differences between these different approaches, and why my third distinct alternative is required.

### Higher-level theories (higher-order thought; global work space theory)

The key attribute of higher-level theories for our purposes is that they do not situate visual experience in the visual cortex (V1–V6). Classically, the distinction has been thought of in terms of whether the neural correlates of consciousness are in the front or the back of the brain (Boly *et al.* 2017; Odegaard *et al.* 2017; Liu *et al.* 2019). The prefrontal cortex (PFC) has become the site of visual experience for higher-level theories of visual consciousness (Frith 2019), including global workspace theories (Dehaene and Naccache 2001) and higher-order thought theories (Lau 2019).

### Mid-level theories (recurrent processing theory; integrated information theory)

Mid-level theories typically attribute visual consciousness to recurrent processing within the visual cortex (V1–V6), either as the basis of their account (recurrent processing theory; Lamme and Roelfsema 2000; Lamme 2018) or because recurrent processing is thought to reflect information integration (integrated information theory; Tononi and Edelman 1998; Oizumi *et al.* 2014). Given the importance of recurrent processing, we might also include predictive coding accounts discussed above (Rao and Ballard 1999).

I will focus on a subset of these theories that explicitly suggest that V1 may be the site of visual experience, including Stoerig and colleagues (Stoerig and Cowey 1995; Stoerig 2001), Pollen, in limited circumstances (Pollen 1995, 1999, 2003), Lamme and colleagues (Lamme *et al.* 2000; Lamme and Roelfsema 2000; Lamme 2003, 2004, 2006, 2018, 2020; Fahrenfort and Lamme 2012); Tong (2003, 2009); Watkins *et al.* (2006); Rees (2007; cf. Rees *et al.* 2002); see Boly *et al.* (2017) for a recent defence.

### Low-level theories (my feedforward account)

Where I differ from these mid-level theories of visual consciousness is that they are unanimous in requiring feedback from higher visual areas to V1 for conscious visual perception (Lamme 2018). As Oizumi *et al.* (2014) note of both recurrent processing theory and information integration theory: ‘The idea that “feed-back”, “reentry”, or “recursion” of some kind may be an essential ingredient of consciousness has many proponents’. By contrast, the essence of my account is that recurrent processing from higher visual areas to V1 is not necessary for visual consciousness.

This is in keeping with mouse physiology where there are no corollaries for the higher visual areas required for such recurrent processing.

It is also in keeping with V1 as a cognitive map. On this account, as we have emphasized, feedforward visual processing in layer 4 reflects our visual experience (V1 as visual map), whilst feed-back visual processing from higher visual areas reflects cognitive processing (V1 as cognitive map). Which of these two approaches is correct? I will take Victor Lamme’s work (Lamme 2020) as my target and highlight three key distinctions between our theories. First, both Lamme (2020) and I (Linton 2017, Ch.1) agree that all leading theories of visual perception (including his) are ‘inferential’, but this is exactly what (from a theoretical perspective) I want to reject. Second, advocates of recurrent processing equate visibility with detectability, but I argue that this leads us into a paradox. Third, Fahrenfort and Lamme (2012) and I disagree as to what constitutes a good paradigm of ‘visual perception’. They focus on illusions, whilst I argue random-dot stereograms, which were favoured by the early vision literature of the 1960s-80s, but have recently fallen out of fashion, teach us the most about the mechanisms of visual experience.

## Vision without inference

Lamme (2020) rightly identifies ‘inference’ as essential to all leading theories of vision (by inference I mean to include Lamme 2020‘s ‘categorizations’ and ‘interference’; cf.; Albertazzi *et al.* 2010, who reject a narrower sense of ‘inference’—‘inverse optics’—than Lamme 2020 and I are discussing). What I propose in Linton (2017), Ch.1 is a theory of vision without inference. By inference I mean the visual constancies, Gestalt groupings and the attribution of meaning to the retinal image. So just as we can distinguish between low-level, mid-level and higher-level theories of visual processing (Cavanagh 2011), we can distinguish between low-level (non-inferential), mid-level (recurrent processing theory; integrated information theory) and higher-level (higher-order thought; global workspace theory) theories of visual consciousness.

The essence of my account is that vision is not about anything. Vision is not trying to ‘represent’ anything, ‘predict’ anything or ‘convey’ anything. Perception is not involved ‘information processing’ or making ‘estimates’ about the external world (Neisser 1967; Miller 2003), much less is vision, or visual processing, in some sense ‘predictive’ (Frith 2013; Clark 2013). Instead, to borrow from a long tradition of naïve realists, I insist (Linton 2017, 74) that vision is ‘silent’, it makes no claims or representations (Austin 1962; Travis 2004, 2013). However, the claim that vision is ‘silent’ is not just the preserve of naïve realism. I develop my own account as a form of naïve or perceptual idealism (Linton 2018a, 2018d), and Papineau (2021) advances a similar claim from a sensationalist perspective. This is very different way of thinking of vision from Marr (1982)’s suggestion that ‘vision is the *process* of discovering from images what is present in the world and where it is.’

The question whether we can account for all of our visual experience without invoking ‘inference’ is an empirical one. First, as I outline below, we need to determine whether a process is properly categorized as ‘perceptual’ or ‘cognitive’. Second, if it truly is perceptual (as, for instance, I argue that depth from binocular disparity is), can it be accounted for in non-inferential terms (for instance, my argument that binocular depth is the product of rivalry eradication, rather than the visual system trying to estimate scene geometry).

## Detection ≠ Perception

A key argument in favour of recurrent processing being necessary for visual consciousness is the suggestion that V1 activation on its own is subjectively undetectable and therefore invisible (Lamme *et al.* 2000; Koivisto and Revonsuo 2010; Koivisto *et al.* 2010; Lamme 2020). Lamme *et al.* (2000) point to the fact that subthreshold changes in colour and luminance are processed in V1 even though they are ‘objectively’ invisible to the observer. But underpinning this suggestion is the assumption that just because a change is not detected it is not perceived. And I argue that this is wrong. Let me explain why this is a shortcoming of signal detection theory.

Consider someone doing a contrast detection task being shown a series of stimuli in succession, one after the other. Imagine the stimulus is a luminous display that takes up their whole visual field. First, they are shown the display with one luminance. Then, this is replaced (without an interval) by a second stimulus where the display is now lighter than the first. But the participant cannot detect that the second stimulus is lighter. Does this mean that the luminance difference between the first and second stimulus is not apparent in their visual experience? Signal detection theory suggest so. But this leads to the following paradox. Now the second stimulus is replaced (without an interval) by a third stimulus that is lighter than the second stimulus, but again the difference is not detectable. And the third stimulus is replaced (without an interval) by a fourth stimulus that is lighter than the third stimulus but again the difference is not detectable. And so on. Clearly the observer’s visual experience is changing. For instance, had the first stimulus been replaced by the fourth stimulus the observer would have immediately noticed. But according to signal detection theory these changes are not just undetectable but invisible (i.e. they have no effect on our visual experience). And we argue this is wrong. The observer’s visual experience will change even though none of the individual changes were detectable. And yet this is the criterion of ‘invisibility’ that recurrent processing theorists employ.

A similar example would be gradual changes, e.g. someone who fails to detect the change in position of a clock hand over time. And a succession of small but undetectable changes is essentially what gradual change-blindness is. Simons *et al.* (2000) and Auvray and O’Regan (2003) suggested that these gradual changes are not only undetectable but also imperceptible (i.e. they’re subjectively invisible). We maintain that the same logic illustrates why this cannot be the case. Imagine that a region of the picture is gradually changing from its initial red to blue. We argue that it cannot really be maintained that the observer’s visual experience remains its initial red over the course of the transition. Instead, a series of subthreshold changes must be changing the observer’s conscious visual perception (Linton 2017, p.102, 2020, p.3189).

Note that this is a different point from Lamme (2003, 2004)’s and Block (2007)’s argument that sudden (masked) ‘change blindness’ is not in fact blindness, but merely ‘inattentional inaccessibility’. Their argument rests on there being a failure of attention in the masked (sudden) ‘change blindness’ case. But gradual change blindness (sub-threshold failures of detection) are not due to failures of attention. As O’Regan (2011) notes, ‘ “you” may even be looking directly at the thing that is changing and still not discover it.’ Instead, ours is the very different point that detection thresholds in signal detection theory provide us with no justification for claiming that sub-threshold stimuli are not consciously experienced.

The same logic applies to the insistence that feedforward processing in V1 cannot be conscious because when backwards masking is applied to halt recurrent processing, such feedforward processing is undetectable. Lamme (2020) terms this ‘objective’ invisibility. But whilst Lamme (2020) suggests that sudden (masked) ‘change blindness’ (which he argues is consciously experienced) ‘is clearly different from invisibility from masking’, we have been given no reason, apart from behaviour, to conclude that backwards masking extinguishes vision in one context (V1 processing without recurrent processing), but merely working memory in the other (sudden change blindness).

## Competing visual paradigms

Finally, Fahrenfort and Lamme (2012) and I disagree over what constitutes a good paradigm for visual processing, and therefore, over what is a good stimulus to test whether recurrent processing is necessary for visual processing. Fahrenfort and Lamme (2012) use three different types of illusions in their ‘no report’ paradigm: (i) pictorial size constancy in the Ponzo illusion, (ii) illusory contours in the Kanizsa triangle, and (iii) the checker shadow illusion. However, I disagree that any of these three illusions are really perceptual.

### Pictorial size constancy in the Ponzo illusion

First, as we have already discussed above in the context of pictorial size constancy (see the discussion of Fig. 3 in ‘Size constancy in pictures’), it is not at all clear that the apparent effect of perspective cues on perceived size is actually distorting visual space as opposed to merely our ability to make accurate judgements about visual space.

### Illusory contours of the Kanizsa triangle

Second, whilst the illusory contours of the Kanizsa triangle are reliant on top-down processing from V2 (Qiu and Von Der Heydt 2005), we argue again (Linton 2017, p.11) that there is a little reason to suppose that these illusory contours are visually perceived. Indeed, how do they manifest themselves in our visual experience? It is sometimes suggested that the contours are seen as lighter than the surrounding. But there seems to be little evidence that this effect can cancel out an actual physical decrease in luminance of the contours, which would be required to show that this really is a perceptual effect.

### Checker shadow illusion

The checker shadow illusion (Adelson 1995) is heavily reliant upon pictorial cues for its effect. Given our argument (in Linton 2017) that pictures and pictorial cues are merely cognitive in nature, we would also argue that such luminance constancy effects are also purely cognitive. The same thought applies to colour constancy, which explains why we do not find Block (1998)’s critique of V1 as the site of visual experience based on the land effect (colour constancy) determinative.

### Random-dot stereogram

What, then, is an appropriate paradigm for thinking about visual perception? This is the topic of the recent debate between myself and Morales, Bax, & Firestone (Morales *et al.* 2020; Linton 2021a; Morales et al. 2021; Morales and Firestone 2021). Morales *et al.* (2020) are concerned with everyday viewing conditions and suggest that visual perception provides us with both the ‘distal stimulus’ (representation of physical 3D world) and the ‘proximal stimulus’ (representation of the retinal image). There is a long tradition of equating one eye vision with the retinal image (‘local signs’: Lotze 1846; Hering 1879; 19th century optometry: Atchison 1979; Johnson *et al.* 2011), which formed the basis of early investigations into the retinotopic nature of V1 (Inouye 1909). And since I argue that pictorial cues are merely cognitive, I am very sympathetic with the idea that monocular vision is just our retinal image. In Linton (2017), Ch.4 I explored the possibility that defocus blur might import some monocular depth (following Zannoli *et al.* 2016), but I am now inclined to think blur is merely a pictorial depth cue, and so all monocular vision is flat in Sacks (2006)’s sense.

However, the argument in Linton (2021a) is that ordinary vision with two eyes doesn’t provide us with our retinal image, nor does it provide us with a representation of the physical world. Instead, what matters is how the visual system resolves binocular disparity (the differences in the retinal images of the two eyes) to give us a single coherent percept. I therefore suggest that viewing a random-dot stereogram in complete darkness and isolation is the appropriate paradigm through which to begin to understand vision. In this regard, I appeal to an older tradition (that was dominant in the 1960s-80s) of ‘basic’ or ‘early’ vision (Julesz 1960, 1971, 1986, 1995; Bishop and Pettigrew 1986; Papathomas *et al.* 1995).

What are the basic components of seeing a random-dot stereogram? You might think it has two components. First, its distance. Second, its shape. But we have argued that its distance is not visually conveyed to us above in ‘V1 as a Cognitive Distance Map’. So all that is left is the perception of 3D shape with its visual scale being ambiguous (what I call ‘phenomenal geometry without scale’ in Linton 2017).

**Figure 4.**
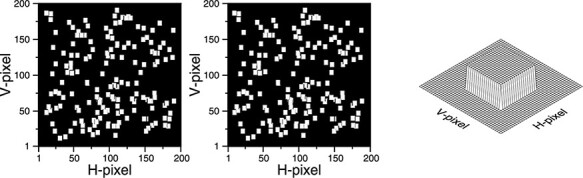
Example of a random-dot stereogram from Serrano-Pedraza *et al.* (2016), and an illustration of the shape it produces when the right eye sees the left image and the left eye sees the right image (‘cross-fusion’). © Serrano-Pedraza *et al*. (CC-BY-4.0).

Turning to 3D shape, and consistent with my suggestion that vision is ‘silent’, I don’t believe stereo vision is a process of trying to estimate the scene geometry of the world from the different view points of the two eyes. Indeed, trying to do this would be meaningless without the vergence signal (Wallach and Zuckerman 1963), which is exactly what we have illustrated the visual system does not have. Instead, I think the most plausible explanation for stereo depth is that it is the solution to a very different problem that has nothing to do with 3D shape. And that problem is binocular rivalry. The percept we see in Fig. 4 is the only percept we could see if we are to eradicate binocular rivalry. So on this account depth from disparity is not about 3D shape, but merely the eradication of binocular rivalry (Linton, in prep). 3D depth is the solution to a problem, not a problem to be solved itself. Specifically, there are no inferences about the 3D geometry of the scene involved.

Strictly speaking I do not think we should even talk about perceiving 3D shape or 3D geometry, as opposed to simply 3D depth (which is merely the resolution of rivalry). Take the following random-dot stereogram from Linton (2021a) (Fig. 5). The process of rivalry resolution I am describing is the process of locating each individual point in depth. This is the same process whether the points make up a single surface (as they do here) or are completely unrelated (as they would be in a random-dot field). That we interpret the points in Fig. 5 as making up a single surface seems to be a cognitive inference based on Gestalt groupings. But all our visual experience conveys to us is individual dots located in a 3D space.

### Motion perception

One criticism of this approach is that it focuses on static binocular vision. Surely motion perception is perceptual, and that involves areas beyond V1 like middle temporal visual area (MT). However, we argue that motion perception is purely cognitive.

First, the fact that the ‘double drift’ motion illusion relies on the PFC is taken as evidence that motion perception, and therefore perception in general, supports a higher-level theory of visual consciousness (Liu *et al.* 2019). However, the illusion does not affect saccadic eye movements (Lisi and Cavanagh 2015; cf., 2017). Either our eye movements don’t act on what we see or, as we argue instead, eye movements provide a more immediate gauge of our visual experience, divorced from the cognitive biases that illusions introduce to the perceptual judgements and more deliberative actions tested by Lisi and Cavanagh (2017) and Liu *et al.* (2019).

Second, motion perception is really just the integration of extinguished percepts over time. In Linton (2017), pp.58–61, we ask why this process is presumed to be perceptual rather than cognitive? For instance, we see apparent motion between two dots blinking one after the other (‘beta motion’; Boeykens *et al.* 2020). But take one of these dots blinking in isolation. It is not at all clear that the judgement that the dot is ‘blinking’—which relies heavily on working memory to integrate percepts over time—is perceptual rather than cognitive. But if that’s true of one single dot blinking, why should not it be true of motion between the two dots?

Third, blindsight often claims to be a perceptual judgement, without visual experience, that something is moving in the visual field (Barbur *et al.* 1994; Zeki and Ffytche 1998; Azzopardi and Hock 2011). Whether blindsight exists in this sense or is merely degraded visual experience, continues to be debated (Azzopardi and Cowey 1997; Overgaard *et al.* 2008; Weiskrantz 2009; Phillips 2020). But the idea that motion perception is merely cognitive remains a distinct possibility here, and if so, may provide insights for ‘normal’ motion perception.

## Objections

Finally, let us turn to four potential objections reviewers have raised with my account.

### Is this a theory of visual consciousness?

Whilst Section 3 provides a theory of the neural correlates of visual consciousness, the argument is that Section 4 is not yet a theory of visual consciousness because it doesn’t ‘explain why’. First, it does not explain why we experience visual consciousness (the ‘hard problem’ is unresolved; Chalmers 1995). Second, it does not explain why we experience cognitive phenomenology in some contexts (e.g. reading) but not in others. In reply, I would argue that consciousness science today is very much akin to astronomy in the 17th century. It is important to remember that there were accounts that ‘explained why’ the planets moved based on magnetism (Gilbert 1600) and vortices (Descartes 1644). But we made progress by embracing a theory that did not attempt to ‘explain why’ (Newton 1687: ‘I feign no hypothesis’), even though this failure to ‘explain why’ left Newton open to the objection (from Leibniz in Clarke 1717), that this rendered planetary motion ‘inexplicable, unintelligible, precarious, groundless, and unexampled.’

### What is pure visual consciousness without cognition?

I try to address this with my discussion of random-dot stereograms viewed in isolation and in darkness. With single eye viewing, there is nothing to suggest that we experience anything different from our retina image. What we see is flat. We know this because if we introduce a second identical retinal image from the other eye (zero disparity), our visual experience will not change, and zero disparity stimuli are flat (I think it’s helpful to think of single eye viewing as akin to zero disparity viewing). The random-dot surface is some indeterminate extension away from us, but I argue that it would be a category mistake to attribute any physical distance to this phenomenal extension:

“When we watch a movie, the movie is always at the same fixed phenomenal depth: the screen never appears to move towards us or away from us. And yet the fact that the movie is always at the same fixed phenomenal depth in no way impedes our enjoyment of the film: the movie can transition from wide-panning shots of a cityscape to close-ups of a face without this transition being jarring. Attributing a variety of different scales to scenes viewed at a single fixed phenomenal depth is therefore something that seems natural to humans.” (Linton 2018e)

The difference between monocular (one eye) and binocular (two eye) viewing is the sense of depth we perceive with two eyes, which is experienced in a random-dot stereogram when there is disparity (a difference in the retinal images). But this, I argue, is simply the outcome of the visual system resolving binocular rivalry. It moves visual points in depth (off the focal plane) so that they do not rival each other anymore. There is no inference involved.

### How do we determine whether an effect is perceptual or cognitive?

For any other potentially perceptual process, it is an empirical question whether it is really perceptual or cognitive. We have to ask two questions.

First, how does the process affect our visual experience? For the Kanizsa triangle the illusory contours are said to look brighter. For the Müller–Lyer illusion, lines are meant to look longer/shorter. For vergence, objects are meant to look smaller and closer. The key point being that you have to be able to point to something in our visual experience that changes. It cannot just be ‘a feeling’ [see my disagreement with Knotts *et al.* 2020 in ‘Perception/cognition distinction’ above]. This is why I resist the suggestion that reading is perceptual. What changes in our visual experience?

Second, to test whether any apparent change in our visual experience really is a change in our visual experience, you need to devise an experiment that shows that this effect persists where a purely cognitive effect would not. With vergence I showed that this was not the case by retesting vergence as a size and distance cue whilst removing any subjective knowledge about the change in vergence and showing that changes in vergence had no effect on our visual experience.

### Visual consciousness in the absence of V1

Silvanto *et al.* (2007) and Koivisto *et al.* (2010) document how neural plasticity can give rise to visual experience when V1 is no longer intact. Does this indicate that V1 is not actually necessary for visual experience? In a literal sense, yes. But I would make two points.

First, Ajina *et al.* (2015) illustrate that when V5/MT+ provides the basis for visual experience in these patients, it does not represent ‘ordinary’ V5/MT+ functioning, but instead appears to take on the functions of the missing V1. And, as we saw above, it appears that V1 itself can be recruited for other modalities with extended visual deprivation. How the brain can change a neuron from one experience to another (in the case of V1 recruitment) is essentially as puzzling as the hard problem itself (why any neuron should give rise to experience, and just as importantly, that specific experience). But I think what this literature shows is just how flexible the brain is at allocating functions, as opposed to saying that the functions associated with V1 feedforward processing cannot give rise to visual experience.

Second, the fact that feedforward processing from LGN to V5/MT+ gives rise to visual experience (Ajina and Bridge 2018), in much the same way that feedforward processing from LGN to V1 does, is actually supportive of my account, since there seems to be little prospect of the recurrent processing necessary for recurrent processing accounts.

## Conclusion

One approach to visual neuroscience is to take a perceptual phenomenon that is already relatively well understood and to try and find the neural basis for it. But another approach is to take a neural mechanism that is already well explored and to argue for a new interpretation of it. For instance, Rao and Ballard (1999)’s initial argument for predictive coding rested on a reinterpretation of surround suppression found by Hubel and Wiesel (1965) (see Keller and Mrsic-Flogel 2018). In this paper, we take two neural mechanisms that have been relatively well explored over the last three decades, the extraction of the distance from vergence and the scaling of the retinal image using vergence, and argue for a reinterpretation of these basic visual functions as cognitive instead of perceptual. Given the interaction betweenvergence and multisensory integration in the Taylor illusion, as well as failures of mandatory fusion in multisensory integration, I extend this account to multisensory integration as well. We argue that the finding that such basic functions as the processing of size and distance in V1 is cognitive should have significant implications for our understanding of V1, not just as an egocentric cognitive map, but also as the site of a low-level theory of visual consciousness.

## Data Availability

Data for Linton (2020) is available at https://osf.io/2xuwn/ and data for Linton (2021b) is available at https://osf.io/5nwaz/.
